# Investigating Philosophies Underpinning Dietetic Private Practice

**DOI:** 10.3390/bs7010011

**Published:** 2017-03-01

**Authors:** Claudia Harper, Judith Maher

**Affiliations:** 1 The Boden Institute of Obesity, Nutrition, Exercise and Eating Disorders, Sydney Medical School, The University of Sydney, Level 2, Charles Perkins Centre, John Hopkins Drive, Camperdown, NSW 2006, Australia; 2 Faculty of Science, Health, Education and Engineering, University of the Sunshine Coast, 90 Sippy Downs Dr, Sippy Downs, QLD 4556, Australia; jmaher@usc.edu.au

**Keywords:** grounded theory, health care professionals, diet therapy, knowledge utilization, philosophy, relationships, patient-provider, theory development

## Abstract

There is limited theory or knowledge regarding dietitians’ practice philosophies and how these philosophies are generated and incorporated into their professional practices. For the purposes of this study, a conceptual framework will explain and define the ‘philosophies’ as three different types of knowledge; episteme, techne, and phronesis. This study aimed to develop an explanatory theory of how dietitians in private practice source, utilise, and integrate practice philosophies. A grounded theory qualitative methodology was used to inform the sampling strategy, data collection, and analytical processes. Semi-structured interviews with dietitians in private practice were undertaken and data were collected and analysed concurrently. The results show that dietitians form collaborative relationships with their clients, in order to nurture change over time. They use intrinsic and intertwined forms of episteme, techne, and phronesis, which allow them to respond both practically and sensitively to their clients’ needs. The learning and integration of these forms of knowledge are situated in their own practice experience. Dietitians adapt through experience, feedback, and reflection. This study highlights that private practice offers a unique context in which dietitians deal with complex issues, by utilising and adapting their philosophies.

## 1. Introduction

The profession of dietetics, consistent with medicine, nursing, and other allied health care providers, is firmly based in ‘Evidence-Based Practice’ (EBP). Sackett et al., the forefather of EBP, stated that EBP is the “conscientious, explicit and judicious use of current best evidence in making decisions about the care of individual patients” [1] (p. 71). The rationale behind EBP, which originated in the medical field, was to increase patient safety and to ensure that the best known intervention was being employed [1]. Health practitioners and their auditors feel confident that, when using EBP guidelines, the applied therapy is based on sound theory and evidence. In Australia, the accrediting professional body/organisation for Dietitians, the Dietitians Association of Australia (DAA), explicitly requires that EBP is used [2,3].

The DAA and the International Confederation of Dietetic Associations (ICDA) states that dietitians need to utilize EBP in conjunction with both the “dietitians’ expertise and judgment, and the clients’ or communities’ unique values and circumstances to guide decision-making in dietetics” [2,4]. This is particularly true for dietitians in private practice; an area of practice which has been poorly researched, despite the rapidly growing numbers in this sector in recent years [5]. In addition, although encouraging a practice that integrates clinical judgment and patient values with EBP, accreditation requirements for dietetic university programs heavily emphasize a biomedical reductionist model of practice [6]. Despite these codes of practice, there is no description of the level of expertise needed to effectively carry out this practice and there is little knowledge of whether or how dietitians should do this in practice [2,4].

Nutrition and food behavior is a complex area with many competing influences, posing challenges to the formulation and application of applicable EBP. Such influences include, but are not limited to: ‘taste and food preferences, weight concerns, physiology, time and convenience, environment, abundance of foods, economics, media/marketing, perceived product safety, culture, and attitudes/beliefs’ [7].

While there are no questions about whether or not EBP works under the conditions that the research was pursued, there is a realization that these conditions are rarely reproduced in individual circumstances [8]. Additionally, evidence based on a single intervention is not congruent in a clinical setting, where a range of treatment approaches are often required [8]. Other health modalities have also noted that EBP constrains practice to quantifiable aspects and that there are areas within the patient-practitioner relationship that have been overlooked.

Perspectives and studies on exerting change in nutrition behavior have been primarily generated in the public health sector and, as such, possible problems and solutions have also largely been discussed in this context. On a public health scale, EBP has failed to exert meaningful change in the health behavior of targeted populations [9]. Buchanan suggests this could be due to social science attempting to apply a natural science perspective to the complexity of human behavior [9]. In effect, natural or biomedical science cannot adequately study, explain, or address phenomena in which the subjects have volition to choose their own actions [9]. Others call for more social research to explore the areas that are poorly understood when it comes to applying successful interventions within the social and environmental contexts of nutrition and health behavior [10,11].

Schubert et al. and Buchanan suggest that the analytical approach to nutrition science fails to recognize diet and nutritional disease as a complex set of processes outside of predictable scientific models [9,10]. Further, research is greatly removed from the human level, where dietitians interact with their clients and their dietary practices within the consultation process [10]. Studies that have explored nutrition and dietetic consultation practices generally focus on dietitians working in the hospital or community health arenas, and on singular aspects of practice. Some of these aspects include verbal and non-verbal communication skills [12], trust in communication between dietitians and clients [13], patients’ experiences of dietetic consultations [14], dietitians use of EBP [15,16,17], and dietitians’ opinions of client-centered nutrition counselling. Most studies investigate what dietary interventions are needed to effect nutritional change; however, less attention is paid to counselling approaches in dietetic consultation [14]. To date, no studies have explored the knowledge types in relation to nutrition and dietetic practices.

Proponents within medicine and nursing have noted gaps in research where EBP has failed to address important areas of practice that exist on the human level, in consultation with the patient [18,19,20,21]. They have proposed a practice framework which incorporates three philosophies that arguably underpin the skillsets and behaviors of practitioners whom are able to practice in a holistic way, working to address the particular needs of the patient [18,19,20,21,22,23,24]. These philosophies encompass reflective practice and clinical judgement, as well as skills that are not easily identifiable in a quantitative way [18,22,24,25]. These philosophies; episteme, techne, and phronesis, are identified as types of knowledge and originate from Aristotle’s Nicomachean Ethics [26].

Practitioners who possess the knowledge types episteme and techne are said to be competent practitioners. However, those practitioners who possess phronesis, in addition to episteme and techne, utilize knowledge and skillsets that show expert characteristics [25,27,28,29]. It has been shown within nursing, that practitioners exhibiting the use of these three concepts, are better able to respond contextually and appropriately to their patients [30,31]. Others have noted that practice and research which incorporates phronesis with episteme and techne, may help bridge the gap between theory and practice, and encompass knowledge and skills that are not effectively conceived within a biomedical science paradigm [18,21,23,29].

Episteme, translated to epistemology, refers to ‘the branch of philosophy which investigates the origin, nature, method and limits of human knowledge’ [32]. In essence, episteme encompasses what is seen as true scientific knowledge based on evidence, which is the basis of dietetic practice. The term epistemology has come to be understood as scientific knowledge which is universal, invariable, and context independent. The most highly respected form of scientific knowledge in healthcare comes from randomly controlled clinical trials, in which theories are tested and found to be true or untrue.

Techne denotes production, namely art, workmanship, or skill (the ‘know how’). It describes the endeavor of using technical rationality to produce a certain outcome [28]. Technical rationality describes a practice of systematically performing a set of discrete tasks, in order to produce a generic product [28]. This ensures that the practice is efficient, standardized, and ‘practitioner proof’ [28]. In this way, it can be replicated and controlled, and lends itself to practice criteria that are easily assessed and accounted for [28]. This aligns with clinical reasoning in medicine, in that, decisions made objectively whilst remaining neutral and which are based on the strongest evidence, should produce results free from conflict; with respect to multiple values and interests [28]. By utilizing both episteme and techne in practice, evidential knowledge is combined with a good technique. However, relying on technique alone may be inappropriate within complex contemporary situations [29]. Expert judgement in contextual situations requires practical judgement, and phronesis helps fill the gaps that good technique alone cannot address [21,29].

Phronesis is most often translated as ‘practical wisdom’ or prudence, and denotes the ability or character trait of being able to use one’s collective knowledge in a different way, in order to produce the most optimal outcome of a specific situation [32]. A phronetic practitioner is able to draw on their knowledge, recognize what is needed in that situation, and deal with it effectively [28]. This ability requires a high level of perception and flexibility, in combination with an understanding of which epistemic and technical knowledge to apply for the best outcome [28]. Benner et al. state that phronesis is necessary to evaluate and integrate evidence and techne [18]. Phronetic action is not easily formulated as it requires creative insight and the ability to actuate knowledge with appropriateness to the situation [18,28]. Phronesis is concerned with the highest good in a situation and requires moral deliberation [33]. Phronesis is dependent on experiential learning, is changeable, and develops in practical endeavors [18,33]. It is also described as being ethical and reflective in practice, and develops through interactions with others [29,34]. 

Based on the above, our aim was to investigate which forms of knowledge dietitians use, and how these are developed and incorporated within the practices of dietitians in private practice. We are using Gustavsson’s description of knowledge to guide us: “Knowledge is not merely theoretical, it is also practical, it is about what we know, what we do and how we act” [35].

In this text, we alternate between the use of the terms, patient and client, as well as, participant and dietitian.

## 2. Method

Constructivist grounded theory, developed by Charmaz, was used to explore what types of knowledge dietitians use in private practice in Australia [36]. How these knowledge philosophies are acquired, developed, and integrated into practice, was also investigated. Consistent with Blumer and Charmaz, sensitizing concepts were used to shape the research topic and guide the questions [36,37]. We used Aristotle’s philosophical concepts of knowledge to provide us with a starting point for our investigation. These three types of knowledge, stemming from Aristotle’s intellectual virtues, are: episteme, techne, and phronesis [38]. Our awareness of these knowledge philosophies assisted our discernment between diverse types of knowledge, despite their acquisition and use being simultaneous and interconnected.

It is important to note, however, that these concepts were not used to force the data into preconceived perspectives. According to Charmaz, researchers using constructivist grounded theory construct theories through interactions, perspectives, and practices [36]. Charmaz explicitly states that theories formulated by researchers using constructive methodology, are an interpretive description of the phenomena under study [36]. This study was approved by the University of the Sunshine Coast Human Ethics Committee.

### 2.1. Recruitment and Sampling

Accredited Practicing Dietitians (APD) in private practice, registered with the Dietitians Association of Australia (DAA), were identified via the DAA website. The DAA is the regulatory body for dietitians in Australia. It is a requirement that practicing dietitians register with the DAA, in order to gain APD status. Dietitians may then be listed on their website and searched for by the public. It is assured that all dietitians listed as an APD on the DAA website hold the credentials to practice as a dietitian.

Invitation emails were sent to 89 dietitians listed as APDs on the DAA website, inviting them to take part in the study. The first round of dietitians were chosen randomly and, as analysis progressed, the recruitment of dietitians became more specified, with the aim of testing emerging concepts within broader sample characteristics. To add robustness to our categories, once we had noted similar concepts within less experienced dietitians, more experienced dietitians were targeted, in order to expand our sample. For example, when a theme of dietitians experiencing a ‘fast learning curve’ and then a levelling out of this learning, emerged in dietitians with less than five years of experience, dietitians with more than 20 years experience were asked whether they remembered a similar process. As a result, an additional theme of ‘lifelong learning’ emerged in those dietitians with more experience.

Eleven dietitians, registered as APDs at the time of this study, agreed to participate in the study. This represents a 12% response rate. The length of time that had been spent working as a dietitian in private practice spanned between seven months and 30 years. All but one dietitian was self-employed. Two dietitians included in the sample were male. The dietitians in this study were a mixture of generalist and specialist dietitians, and two dietitians held double degrees, with the additional degree being held in another allied health modality. One dietitian held a Diabetes Educator status.

### 2.2. Data Collection and Analysis

Data were collected via semi structured interviews with dietitians, who responded affirmatively to invitation emails. A pilot interview was conducted prior to the commencement of data collection. The pilot interview indicated that the interview format was appropriate for gathering rich data associated with our subject matter. One-on-one, in depth, semi structured interviews were conducted in person (eight interviews), or on the telephone (three interviews). Telephone interviews were conducted to gain access to dietitians who were not close enough to interview in person. This also helped broaden the sample. The interviews were recorded with a recording device and a recording service was used to record the phone interviews. All interviews were transcribed by the researcher verbatim. This process allowed the researcher to become more familiar with the data. 

In the interview, participants were asked to describe an interesting or recent case which they had worked on. The purpose of this was to explore a case that the participants could recollect in more detail. Subsequent questions allowed an exploration of the participant’s thoughts, decisions, and reasoning, before, during, and after the case. At times, the interview process became reflective, due to the participant needing to reflect on why they chose to do things in a certain way, or how they arrived at the decisions that they had made. As data collection and analysis proceeded, and emerging concepts became apparent, additional questions were asked, in order to develop the categories more fully. For example, when data analysis indicated that being a self-employed business owner significantly contributed to the subject matter, this area was explored in more detail in subsequent interviews.

Observations and perceptions made during the interview were recorded as field notes at a convenient time and were used to provide further context on the emerging categories. During transcription and throughout analysis, further thoughts surrounding the data were recorded as memos and used to add depth to the analytical process [36]. 

Consistent with grounded theory, data analysis occurred concurrently with data collection [36]. In the initial open coding phase, preliminary codes were ascribed to words, lines, or incidents within the data. The data were preliminarily open-coded in Microsoft WordPerfect10 (Microsoft, Washington, DC, USA). We then used NVivo10 qualitative software (QSR International Pty Ltd, Melbourne, Australia) to store and group the data. Codes were crosschecked across the data set, to ensure the accuracy of the emerging concepts. Codes that appeared frequently or seemed significant across the data, were focused into categories. 

Theoretical coding, according to Charmaz, conceptualizes possible relationships between the categories that were developed during the previous analytical stage [36]. For instance, the category ‘applying evidence contextually and collaboratively’ subsumed a number of codes that described strategies and information which dietitians used to determine appropriate therapies in practice. One other category, ‘non prescriptive’, encompassed various approaches to nutrition therapy favored by the dietitians. These categories were then encompassed within the conceptual category, ‘collaborative therapy’, as a way in which dietitians make decisions on nutrition therapy. Other major categories, ‘nurturing change’ and ‘evolution and adaptation’, also subsumed various processes and strategies.

Because our area of interest was the types of knowledge that dietitians use, the contextual information detected during this study, along with observational memos, were used to create a model of the knowledge philosophies that underpin the key strategies and skills which dietitians used in this study. This model of private practice also described the contexts and processes within which dietitians acquired, developed, and integrated these knowledge philosophies into practice skills. 

## 3. Results

**Figure 1:**
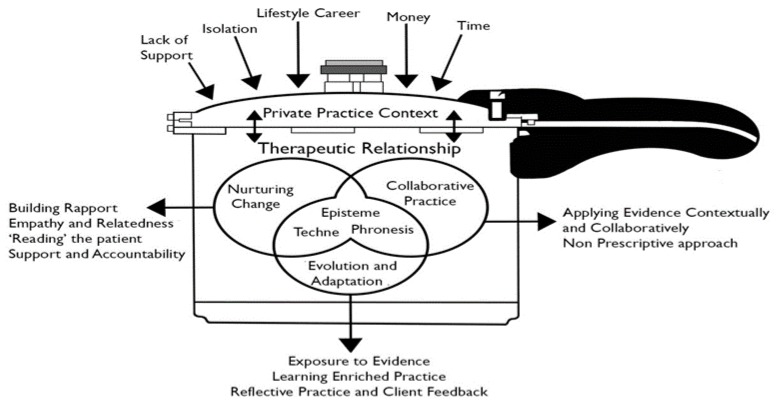
Philosophies Underpinning Dietetic Private Practice.

In the presentation of our results, we will first describe the overview of our theoretical model of private practice, summarized in Figure 1. This model of private practice was generated from the data, as themes which were common across the data set became apparent, and were elevated and organized into concepts and overarching categories.

We will then, in turn, explain each component of the model, beginning with describing the unique context of private practice. We will describe the therapeutic relationships and the individual key strategies and processes that dietitians utilize to foster these in private practice. Finally, we will elucidate the knowledge philosophies underpinning these processes and show how these philosophies are integrated within processes that allow dietitians to adapt and evolve their practices. 

### 3.1. Overview

As depicted in Figure 1, private practice is a complex and pressured environment within which dietitians adapt and evolve their practices. The unique context of private practice (described below) compels dietitians to modify their practices and foster mutually beneficial therapeutic relationships with their patients.

Synergistically, the therapeutic relationships themselves provide a context in which dietitians hone the skills and strategies needed within their practices. The key strategies used by dietitians with their patients: ‘nurturing change’ and ‘collaborative practice’, are continuously being refined using the third strategy of ‘evolution and adaptation’. Underpinning and intertwined within these strategies, the three intellectual virtues: episteme, techne, and phronesis, were evident. These knowledge types remain at the center of this model. This is due to their presence being fundamental to the way in which dietitians adapt and evolve their strategies, in relation to the therapeutic relationships that are cultivated in the context of private practice. We will consider these concepts separately, explaining each of them in turn. Firstly, we will set the scene in the context of private practice.

### 3.2. Private Practice Context

Private practice dietetics appears to be a unique context within which dietitians acquire skills and use knowledge specific to that setting. It presents as a complex and pressured environment, within which dietitians adapt and evolve their practices. Private practitioners experience concerns over money and time, a lack of support, isolation, and enmeshment of their work and personal life. The private practitioners in this study acknowledged the challenging aspects of working in a private practice, which involved many hours of unpaid work, running a business, and doing the necessary paperwork that goes along with being both a dietitian, and being a business owner. Unpaid time was spent on business endeavors, preparing and updating education material, reading, keeping up to date, and professional development. Positive aspects of working in private practice included the freedom to choose which areas of interest they would focus their practice on and being one’s own boss. These positive aspects were more prevalent among more established dietitians. The dietitians in this study indicated that running a business was more suitable for their perceived personalities.

Dietitians in this study spoke of the necessity of entering private practice due to the lack of work for dietitians. The more established dietitians expressed concerns that new graduates are entering private practice with no support or experience, and who are accepting an income well below what they should, thus creating a negative impression of the dietetic profession as a whole. Dietitians expressed that there was a lack of education on the complex aspects of private practice. These aspects included the wide array of conditions encountered, business acumen, people skills needed for dealing with frequently emotional patients, and the psychological aspects of feeling emotionally drained or experiencing death in long term patients with whom close therapeutic relationships have been established.

A sense of isolation from peers was experienced by the dietitians that worked alone. Some spoke of being comfortable with this isolation and had no desire to change. Others had sought out opportunities to connect with other dietitians, to varying degrees. Competition with other local dietitians, in some cases, was expressed as paradoxical to making connections.

The participants expressed the desire to deliver the best service that they could, and were continuously adapting their practice and knowledge base in an effort to better serve their client base. On account of this, dietitians spoke of the all-encompassing nature of being a dietitian in private practice. When they weren’t actively working in their practice, they were learning, reading, and thinking about nutrition and business. In effect, this created a career which could be described as a ‘lifestyle career’, as it was often difficult to ‘turn off’, even when outside of the practice. The high exposure which nutrition, food, and cooking has within society through various outlets, also added to this feeling. Those that also lived in the community which they worked in described the balancing act of seeing clients both professionally and in a more casual setting, and being conscious of the relationship which they had with their clients. Overall, private practice provided a rich context in which dietitians worked and in which they learned the skills which are needed to be successful.

### 3.3. Therapeutic Relationship

The therapeutic relationship is the foundation of the practitioner-patient alliance. Research suggests that this relationship has two parts: building a relationship and facilitating positive action [39]. A successful therapeutic relationship requires the practitioner to build a good rapport, show empathy, and gain the trust of their client [39]. Braude states that within the intersubjective space between the practitioner and patient, elements exist that are beyond the cognitions of the practitioner and patient [20]. Despite being beyond cognition, these nonverbal, tacit clues that exist within the context of the therapeutic relationship, provide valuable information to the practitioner, which they draw upon to appropriately respond to their patient [20].

Dietitians in private practice recognized the importance of developing a therapeutic relationship as the foundation of their practice. They have developed strategies that directly relate to fostering a mutually satisfying therapeutic relationship with their clients. The motivations for dietitians in private practice to foster these relationships are multi-faceted. The participants in this study described the importance of the therapeutic relationship for facilitating change and positive outcomes for their clients. Furthermore, they noted that positive outcomes were more likely in clients who received ongoing support through follow-up consultations over time. Follow-up consultations and the resultant positive outcomes were mediated by the therapeutic relationships that dietitians fostered from the outset of contact. Additionally, dietitians in this study were aware that building these relationships were crucial to their professional reputation with both doctors and patients, and that, in turn, they had a direct effect on their income. In this way, the therapeutic relationship was vital to both the client’s wellbeing and the practitioner’s livelihood.

### 3.4. Collaborative Therapy

Collaborative therapy refers to the key strategy that dietitians in this study used to formulate their education focus and nutrition interventions with their clients. Using a non-prescriptive approach, and applying evidence both contextually and collaboratively, are ways in which the dietitians in this study worked collaboratively with their clients. These processes were the most commonly described approaches across the data set and they are elucidated below.

#### 3.4.1. Evidence Applied Contextually and Collaboratively

Dietitians in this study described working with their patients to design therapies that were informed by EBP, but that were, more importantly, collaboratively formulated with the patient. The clients’ social contexts were often barriers to implementing strict EBPs and necessitated a flexible approach. One dietitian showed awareness of the clinical guidelines for a low salt diet, but access to suitable foods and the clients’ lifestyle prohibited recommending a diet which was strictly based on clinical guidelines:

‘It wasn’t a clinical low salt diet, it’s quite difficult to access that sort of food here unless you’re going to cook absolutely everything from scratch which wasn’t quite realistic for her. We also discussed how to, you know, eat out, get take away in a sensible manner. She had become quite reliant on take away for small periods of time there, she had her children, came back home and was doing lots of events of the evening, so she needed to do that sort of thing so it was about, so if you need to do take away how can we do that well?’

(Dietitian 7)

The primary goal of the dietitians was to ascertain the client’s abilities, requirements, and wishes, and to collaborate with their client in order to tailor a suitable intervention protocol. Facilitating client autonomy within this collaborative process, where possible and practical, was seen by dietitians as a necessary part of gaining trust, enhancing a rapport, and increasing compliance with their clients. They described the approach of telling a client what they had to do as having the potential of alienating the client and getting them off side. One dietitian described a client who arrived with a diet plan that she wanted to follow, and which had previously allowed her to successfully lose weight. The dietitian chose to facilitate this to show support for the client and encourage follow-up visits. Over time, taking into consideration her previous failures, the dietitian hoped to work with the client to make her diet more easily sustainable over the long term:

‘(She) had specifically come to me because she said she wanted some accountability and just a little bit of support along the way so, in many ways I was guided by what she wanted to do. . . given her previous failures, that we would have to look at something that I think would be a bit more sustainable for her. But in the first appointment I decided to let her run with what she wanted to start with because I wanted to show, demonstrate to her that I was, you know, being supportive of what she wanted to do.’

(Dietitian 8)

Evidence applied contextually and collaboratively as episteme and techne. Episteme as a knowledge type underpinning practice is evident in the use of evidence-based guidelines for nutrition interventions and counselling techniques. Techne, as a knowledge type, is present when dietitians have the technical acumen to use this knowledge and apply it appropriately to suit their clients’ needs. In this way, episteme and techne underpin the DAA competency standards which state that, for dietitians to be competent, they must be able to use client care in collaboration with clients.

#### 3.4.2. Non Prescriptive

The dietitians in this study most often used a non-prescriptive diet approach during intervention. Increasing awareness whilst eating and educating to increase nourishment within an often imperfect diet, was seen as more desirable than promoting prescriptive diets. Some others spoke of supplying a diet plan to their clients, when they felt that the client preferred that approach. One participant described how being non-prescriptive worked well for her clients:

‘It’s not prescriptive; it’s them figuring it out for themselves. That’s how I work anyway and it works really well.’

(Dietitian 3)

Non-prescriptive as episteme and techne. Using a non-prescriptive diet for clients would be underpinned by episteme and techne, in the use of evidence which signifies that changes are needed in the diet and using the techniques to apply changes in a non-prescriptive way.

### 3.5. Nurturing Change

Nurturing change refers to the key strategy that the dietitians in this study used to foster an environment in which their clients felt heard and supported. The processes that constitute ‘nurturing change’ are, ‘building rapport’, ‘empathy and relatedness’, ‘reading the patient’, and ‘support and accountability’. These relational and interpersonal skills were the most commonly described processes across the data set. They are individually considered below.

#### 3.5.1. Building Rapport and Relationships

The dietitians in this study were conscious of establishing a good relationship with their clients. They were aware that, prior to any education or intervention being delivered or being successful, they need to establish a relationship in which the client feels comfortable and feels as though they are engaged in the consultation process. The techniques that they use to build a rapport and relationships vary, according to the dietitian and the client.

The dietitians reported changing their approach, depending on the type of client that they had. They use a range of techniques to show the client that they are interested, respectful, and willing to listen. In doing so, they were more confident that the client would be more engaged with them and more likely to return to see them. Repeat visits were seen as much more conducive to successful outcomes for the client, as well as being advantageous for the business. One dietitian spoke of the importance of engaging the client and making them comfortable, in order to be able to help them:

‘I know if I can’t engage that patient that I’m not going to be able to help them so it’s of primary importance to make sure you are on that level with the patient and they’re happy with you, you know, if they’re not happy with you, or comfortable with me I’m not going to be able to help them so I need to kind of establish that right from the beginning.’

(Dietitian 6)

Another dietitian described being aware that more than one consultation is usually needed to help a client achieve positive outcomes, and that facilitating follow-up visits hinged on establishing a rapport and connection, right from the first consult:

‘Generally I try and establish a really good rapport and connection with the person because I know that if I can establish that rapport then I know that I can keep them coming back and I know that I can help them, whereas if we can’t establish that connection, so that’s what I sort of try to establish in that first consult.’

(Dietitian 9)

Building rapport and relationships as episteme and techne. Although not stated explicitly in the competency standards, building a rapport with clients is a fundamental element of counselling models and is implied in dietetic counselling practice. In this way, episteme and techne are evident as knowledge types underpinning the techniques used to a build rapport and relationships when counselling clients.

#### 3.5.2. Empathy and Relatedness

All participants described the amount of emotionality that they encounter in consultations with their clients. Some dietitians felt this emotionality was due to the more personal and unregimented atmosphere of the private practice consultation process. This atmosphere was contrasted with consults in hospital wards by dietitians who had experience in both areas. Depression is often encountered and crying was encountered on a very regular basis by all dietitians in this study. Dietitians in this study felt that being respectful and empathetic during these encounters was crucial. Often, dietitians described crying as flattering and showing trust, and also as cathartic, moving the consultation in a more beneficial direction. All dietitians reported that when the emotionality became counterproductive to moving forward, they would encourage clients to seek professional help. The dietitians in this study recognized the importance of understanding the client from their perspective and being able to relate to their circumstances. The older dietitians also described scenarios in which having personal experience helped them relate to their client’s situation. The younger dietitians revealed how they had grown more comfortable with clients’ emotions over time. Anger was also encountered, although to a much lesser extent, and was usually dealt with by listening to the clients’ concerns and reassuring them that they are there to help. A dietitian describes becoming comfortable with dealing with crying patients over time:

‘I go through many boxes of tissues in a week, but it’s a tough situation and I remember when I first had my client cry on me, I didn’t know what to do, I didn’t know what to say but I think through experience you get to know it’s part of the whole process—it’s part of that process of change because it’s a realization.’

(Dietitian 2)

An older dietitian described being able to relate to different life stages of her clients, after having the same experiences:

‘I think that it allows you to relate to people at that stage, so when I had young children I could relate to how tired and busy young mums are, when I had teenage daughters I could relate to, you know, so the mother drags the teenage daughter in sits her in the chair and says ‘she eats terribly, fix her diet’ you know, so you can relate to fact that the child is sitting there going, ‘yeah mum I don’t want to be here’ so yeah.’

(Dietitian 4)

Empathy and relatedness as phronesis. Being empathic is a necessary element of being a skillful counsellor [40]. Svenaus (2003) draws a direct comparison between empathy and phronesis, and suggests that empathy is a fundamental component of being a phronetic practitioner who seeks the good for his patient in each situation [41]. In this way, phronesis underpins the practice of being empathetic in the context of this situation.

#### 3.5.3. ‘Reading’ the Patient

The dietitians in this study frequently alluded to ‘picking up’ signals from their clients which helped them decide how to proceed within the consultation. They used a variety of ways to describe this process. These included, ‘gut feel’, ‘intuition’, ‘reading the person’, ‘instinct’, ‘clever questioning’, and ‘tone’. These non-verbal cues or the tone of the conversation, despite being hard for the dietitians to describe, played a significant role in how the dietitians chose to proceed and respond throughout the consultation. During the interviews, the dietitians could recall how the patient was feeling and described asking direct questions, if they weren’t sure.

Most dietitians described this as a natural ability that they had, but which they honed over time:

‘Yeah, I guess I think I naturally can read people pretty well that sort of one area I can adapt the way I speak with people, so yeah that instinct as well—it kind of gets better as you go.’

(Dietitian 2)

Most dietitians described using this perceptive ability to help them make clinical decisions:

‘I think you’ve really just got to tailor everything to the individual and that’s all on gut feel. I don’t know that’s the way I operate anyway—it’s—it is a bit gut feel.’

(Dietitian 1)

Reading the patient and phronesis. Braude suggests that perception connects cognition and consciousness, and that it is important for clinical reasoning [20]. He suggests that these perceptions are present within the therapeutic relationship and contends that, when perception and intuition are combined with the explicit, phronesis is a fitting concept to apply to clinical decisions [20]. Phronesis, then, underpins the practice of using tacit clues which are implicit within the consultation process, to assist in being responsive to the patients’ needs. 

#### 3.5.4. Support and Accountability

Dietitians who saw clients regularly over a period of time, reported that their roles moved more towards providing support and accountability, rather than nutritional advice or changes. These consultations became less structured than the first consultations. This dynamic was the most successful in helping their clients change nutritional and lifestyle habits. Slow, long term incremental changes were seen as being more successful in the long run, and repeat visits gave these dietitians the freedom to more successfully support clients and make necessary changes:

‘For review consultations, subsequent consultations, probably a lot more of my energy would be focused on motivational interviewing, health coaching and supportive counselling, that sort of thing. So it’s gradual changes occurring but I think they’re coming about from her having that accountability of coming in and seeing me every week and that’s her decision.’

(Dietitian 10)

### 3.6. Evolution and Adaptation

Acquisition, development, and integration encompass how dietitians are exposed to knowledge, how they seek it out, and how they learn and integrate information and strategies in their practice.

#### 3.6.1. Exposure

In line with the concept of dietetics in private practice being a lifestyle career, dietitians are constantly exposed to nutritional information from the media, social media, supermarkets, newspapers, and magazines, as well as their own clients. They report that, in addition to these sources, they also seek out information on Google Scholar and access online resources provided through dietetic professional organizations, in particular, Practice Based Evidence in Nutrition (PEN). The DAA website provides registered dietitians with online forum style ‘interest groups’, in which dietitians can discuss various topics relating to nutrition and dietetics. Most of the participants in this study found that these forums were an interesting and valuable source of information, and also participated in these groups. Mentors were also described as being useful and valuable sources of information, with some dietitians continuing to choose mentoring options long after the DAA stipulation of one year. In cases where opportunities to speak to other dietitians were taken or sought out, these contacts were described as very helpful. Professional development is a necessity for all accredited dietitians, and dietitians in private practice most often sought learning opportunities in areas that taught motivational interviewing or other courses that concentrated on the psychological aspects of dietetic counselling, rather than the nutritional aspects. One dietitian described how social media and the interest groups provided a constant stream of information:

‘I like loads of different nutrition and dietetic sorts of pages, and it’s probably the quickest and easiest way along with email to [get information]. I’ve always got social media or my emails up there where the interest group stuff is coming through all the time so, yeah so they probably are the two quickest and easiest ways that I’m sort of updating things all the time.’

(Dietitian 5)

All dietitians used PEN and google scholar as sources of evidence:

‘(The) first port of call would probably be PEN, so the data base associated with the DAA and then I’d probably just go google scholar somewhere like that and start looking up papers.’

(Dietitian 11)

Exposure as episteme and techne. Episteme and techne, as knowledge types, underpin having a knowledge base of evidential information and the technique to search for and discern relevant sources of evidence and information, and integrate them into practice.

#### 3.6.2. Learning Enriched in Practice

The development of sound nutrition and practice philosophies occurred most quickly and thoroughly within the context of the consultation process, with secondary input coming from exposure to other information sources. New graduates entering private practice reported a steep learning curve which levelled out in a matter of months with increased exposure to the consultation process, although all dietitians reported continuous learning and improvement over their career span. Dietitians described that building these skills occurred over time with experience, but that they were also building on pre-existing skills and knowledge that they felt they naturally possessed. All dietitians reported constantly adapting their practice and approach, in relation to the perceived needs of their clients. Over time, this process becomes embedded in practice and becomes intrinsic within the dietitian, permitting them become continuously responsive to the changing environment of private practice. One participant described how, through experience, existing skills improve:

‘I think just through experience, I think you know as an initial dietitian you know you can do everything that an experienced dietitian can do just about but you can’t, you can’t convert that into that kind of people skill connection. You can have people skills but through experience you get to know what people respond to, and I think that’s where it’s definitely built from. I’d like to think that my people skills are really good and have always been really good but you still can’t put that into a consultation situation until you’ve been able to do it many times I think.’

(Dietitian 7)

Learning enriched in practice as phronesis. Phronesis requires experiential learning across time, as is evident in our participants, where knowledge is honed, corrected, or rejected [18]. Phronesis is a knowledge type that is essentially changeable and particular to situations. Therefore, it requires experiential learning, during which it can be improved, corrected, and responsive to real events [18].

#### 3.6.3. Reflective Practice and Client Feedback

Practice philosophies are integrated through the process of reflective practice and client feedback. Reflective practice was carried out via mentoring, and through thinking about their performance and what they needed to change. One dietitian set a specific time each week to reflect on where her gaps in knowledge were and how she could improve them. All the dietitians spoke in a reflective manner during the interviews and they were self-aware in knowing their strengths and weaknesses. They described their character traits and their personality types, and how these fitted with private practice and consultations with clients. The participants also reflected on circumstances with patients, both inside and outside of consultations. They thought about and described how they felt about them, how they thought the patient felt, and at times, how they might do things differently, if a similar circumstance happened in future.

Client feedback was provided in the forms of both physical biomarkers and the observation that clients who are happy with the service they are receiving will return for follow-up visits, and that these repeat clients had better success rates. Physical biomarkers were seen as a secondary source of success to any psychological aspects that had changed positively in the client over time and which helped to ensure the continued compliance of any implemented healthy lifestyle changes. One participant described how reflecting on practice culminated in changing strategies with patients:

‘But I feel it just sort of comes around like, when you really reflect on your consultations and you go—ok well you know once you see 30 people you get an idea about what are some common things that come up with clients...and you sort of reflect on that and come up with some strategies that you know are going to help them.’

(Dietitian 3)

One dietitian reflected on how she would react differently, if a similar situation happened in the future:

‘So I guess that first consult, when I reflect back on it was really, you know, probably faced with somebody else in that situation I wouldn’t have done anything, I would’ve said you know “this is what it is” maybe explained it—off to hospital you go.’

(Dietitian 2)

Reflective practice as phronesis. The participants were able to reflect and respond within the consultation in response to cues, as well as reflect on previous circumstances involving patients. They used these reflective skills to help them respond appropriately, increase self-awareness, and improve future performance. In this way, phronesis, which is aligned with reflective practice, underpinned these actions. Phronesis is said to underscore reflection as a means to guide wise action, assist with navigating the various contexts of practice, and ultimately gain practical wisdom [29].

## 4. Discussion

The theoretical model of the philosophies of dietitians in private practice, integrates the experiences, practices, and perspectives of 11 dietitians working in private practices in Australia. It describes the key strategies and processes that dietitians use in the context of private practice and the therapeutic relationships which were found to be important mediators. It also highlights the underlying knowledge types that dietitians utilize when performing and learning these key strategies. Most studies exploring episteme, techne, and in particular, phronesis, as philosophies underpinning health care, concentrate on medicine and nursing [21,30,31,42,43]; they do not account for the context of private practice or the complexities faced by dietitians when helping patients choose and/or change lifelong eating habits.

The processes that the dietitians used in this study shared similarities with health care and counselling models that are a small part of dietetic student curricula [38,40]. This study supports the accepted notion that the counselling relationship is a fundamental aspect of a successful counselling relationship [41]. We extend and enhance this concept by exploring the processes specific to dietetics, as well as by identifying a philosophical/knowledge construct that may be useful in framing the explicated processes and strategies.

The key strategy, ‘collaborative therapy’, shares similarities with what is generally known as ‘patient or client centered care’ [14,44,45]. Nutrition and dietetics suffer from a lack of definition surrounding client-centered care, as it pertains to nutrition counselling [14,44]. The contextual and collaborative approach for applying interventions in this study was, at times, in conflict with applying a straight forward, evidence-based guideline. Despite a lack of time and skill being the most cited barriers to using EBP, the dietitians in this study described the client’s contextual situation as the main driver, when deciding on intervention strategies ahead of EBP [15,46,47]. This disparity could be due to the longer consultation that private practice affords and the possibility of follow-up appointments affording a slower approach to education and therapy. Barriers cited when applying this contextual approach elsewhere, specifically professional norm and organizational culture, are not present in this study, which highlights the professional freedom that dietitians have in private practice, when delivering patient-centered care [44,48]. This contextual approach to intervention was strongly favored by clients of dietitians in a qualitative examination of client preferences [14]. This study confirms that dietitians in private practice are not only aware of the mitigating factors that affect the food choice and practices of their clients, but also strive to collaboratively develop interventions with these in mind. Hancock et al. (2012) found that most patients favored a non-prescriptive approach, although a small subset preferred a specific diet plan [14]. The dietitians in our study appeared aware of which approach their clients preferred and this is reflected in the strategies which they use in response to these perceived preferences.

Building a rapport was shown to be an important aspect of practice in our study. This finding is consistent with research that shows that building a rapport is important for establishing a counselling relationship [41,42]. The practice of building a rapport helps to build trust in clients, allowing for more positive outcomes and relationships [42]. Hancock et al. (2012) found that dietetic clients cited rapport building as being important in their dealings with dietitians [14]. Further to these findings, our study revealed that the context of private practice provided the motivation to establish a rapport with clients, and a rich learning environment in which to foster the skills to do so.

The practices of dietitians in this study support the findings by Cant and Aroni, who found that dietitians strive to show empathy in their consultations by listening, in order to understand the patient’s situation [12]. This is further supported by Goodchild et al., who noted that empathic moments are common throughout dietetic consultations and that these moments are rarely missed by dietitians [49]. Patients reported a higher satisfaction in consultations where responses to emotional opportunities by dietitians were coded as more empathic [49]. Rogers (1940) contends that demonstrating empathy towards the client is an integral part of the counselling process [45]. The dietitians in this study also used their own experiences to relate to the patient empathically. It is suggested that using one’s own experience to relate to the patient may optimize the empathic response and, in turn, improve the patient’s satisfaction [14]. It is argued that empathy as a character trait is needed, to enable an understanding of the needs and wishes of patients [41]. To use empathy as a phronetic, the practitioner would need to seek to understand and desire ethical solutions that align with the patients’ goals for the most beneficial outcome in that situation [41]. This requires the practitioner to be highly cognizant of the patients’ version of what their own best health and wellbeing is. Further study on this concept within dietetics would be beneficial to ascertain levels of empathetic responses within consultations that take into account the patients’ viewpoint of their own best health outcome.

Our study showed that nonverbal communication featured heavily in the way that dietitians responded to their patients. Further, the participants described using perception or intuition when dealing with their clients, and that perceptiveness was intrinsic to them. Nonverbal communication is largely involved with conveying feelings and attitudes, and is often beyond our conscious awareness [12]. Nonverbal communication may account for 65% of meaning in interpersonal communications [50]. The present study confirms the Cant and Aroni finding, that dietitians are aware of nonverbal communication and place an importance on this being nonthreatening [12]. Intuition and perception in dietitians has not been previously researched; however, research on nurses has recognized its presence in nursing practice. Intuition and perception in clinical practice is a form of understanding or knowing something about the situation or patient, without logical thought processes or being explicitly told by the patient [51,52]. Rew and Barrow, in the reviewing literature, found that intuition was vital for complex decision making [53]. A review by King and Appleton found evidence that nurses used intuition (i.e., gut feelings) to initiate action [54]. Rigid adherence to protocol tended to devalue intuition and perception in clinical reasoning [54]. Feasibly, due to having less procedural and departmental constraints, the dietitians in our study were able to recognize and verbalize that intuition and/or perception played an important role when interacting with their clients.

Dietitians in this study saw successful outcomes by providing long term support and accountability to their clients. Hancock et al. found that, in a community and hospital setting, patients reported that support felt limited, due to the length or frequency of the appointment, or the continuity of the dietitian [14]. In a private practice situation, this scenario is avoided and, as such, the dietitians in this study reported successful outcomes for patients that they had seen, or were seeing, long term, both in biomedical markers and habit changes. This indicates that private practice is well placed to provide successful outcomes to those clients who can access long term private practice dietetic support.

The sources of evidence that participants used in this study are in contrast to Thomas et al., who found that pediatric dietitians had access to Medline, Cochrane, and CINAHL, and that 39% used Medline as their main source of information [17]. Dietitians in this study report using online searches (google scholar), and online resources provided by DAA and DAA interest groups, as their main sources of information when sourcing evidence. This is most likely due to the lack of access that private practice dietitians have to other sources of literature that require payment. There are no studies documenting other types of information that are sourced, such as social media, and this study uncovers the phenomenon of dietitians in private practice being inundated by nutrition and food information.

The newer dietitians in this study reported that, upon entering the practice, their communication and counselling skills increased quickly with continuous consultation experience. This supports Cant et al., who found only a small variation in communication practices of recently qualified and more senior dietitians [12]. Situational or experiential learning is at the core of learning for professionals, and health professional training courses require a period of placement types within their profession before graduation [55,56]. This study supports the concept that skill acquisition for professionals is embedded in practice, and highlights the importance of ongoing exposure to clinical settings by professionals as a means to improve and maintain their skills.

As part of the accreditation process, new graduates must enter a mentoring partnership for one year [57]. Part of that process is reflective by design, as the mentoring partnership requires the graduate to explore his/her practice with the mentor [58]. There is little research on the mentoring partnerships in Australia; however, research has shown that the mentoring partnership can facilitate lifelong learning through supporting the habit of reflective practice [58]. Palermo et al. found that mentor attributes of facilitating trusting relationships and providing effective feedback, were important components of mentoring [59]. This study supports this finding, with dietitians in this study reporting positive relationships and experiences with their mentor, and this positive experience compelled some dietitians to seek ongoing mentoring after the mandatory period was over, throughout their career.

Reflective practice is an essential skill for being an APD and dietitians are expected to autonomously continue this practice throughout their career [6]. Reflective practice allows practitioners to learn from experience. Ajjawi et al. found that self-evaluation and reflection in allied health professionals were important for monitoring and correcting clinical reasoning and communication [60]. Proponents of including phronesis as a necessary element, along with episteme and techne, for a model of good practice, and the traits of an expert practitioner, contend that to be phronetic is to be reflective [18,21,29]. Schon and Benner posit that the ability to reflect in and on practice, and change actions within constant contextual shifts within practice, distinguishes an expert from a technically proficient practitioner [25,61]. Kinsella contends that merely reflecting on practice, such as journaling and blogging, is aligned towards techne, and that to aspire to expertise resultant change and critical judgments must be able to be processed and arrived at within the context of the moment [22]. It was evident in this study that dietitians use high level reflective practice both in and out of the consultation experience to improve their interpersonal skills, clinical judgement, and their business models. This study supports the implications that dietitians continue to reflect on practice throughout their career and use those skills to improve services to their clients. This indicates that private practitioners subscribe to the DAA’s stipulations and agree with the value of reflective practice.

## 5. Episteme, Techne, and Phronesis

This model of private practice revealed that dietitians use intertwining and embedded forms of episteme, techne, and phronesis in their practice and throughout the consultation practice. It was clear in this study that the dietitians used episteme, techne, and phronesis when using key strategies and processes, and that these philosophies also underpinned how the participants improved and integrated these strategies into practice. The key strategies that the dietitians employed, although discussed separately, are used in a simultaneous manner in response to the environment. Similarly, it is not possible to separate episteme, techne, and phronesis as underpinning forms of knowledge skills in practice, although at times, one form of knowledge may be predominant over another, depending on the situation. In line with other studies, elements of practice that are not easily described or assessed were evident when dietitians adapted their approach to clients within consultations, in response to both extrinsic and intrinsic cues. This study supported the use of the concept of phronesis through the dietitian’s practices of perception, empathy, and reflective practice.

Phronesis was useful in exploring the therapeutic relationship that is a complex interpersonal experience where dietitians use tacit, theoretical, and evidential knowledge, to appropriately respond to clients. In this space, dietitians simultaneously reflect, respond, and learn. These responses become intrinsically embedded over time. It is also clear that the therapeutic relationship was a crucial platform for using, learning, and integrating these strategies, and that the context of private practice is an important mediator of nurturing the therapeutic relationship. Episteme, techne, and phronesis as a framework for professional practice, provided a useful lens to view dietetic practice, particularly the contexts and elements of practice that are not easily described, but that are crucial to being an expert practitioner.

## 6. Strengths and Limitations of the Study

The study’s findings reveal the key strategies and underpinning philosophies that dietitians use during private practice. To our knowledge, this is the first investigation of dietitians’ philosophies in private practice and shows a unique perspective of private practice in Australia. A strength of this study is that it reveals the dynamics and intricacies within the consultation process, including facets within the therapeutic relationship and strategies used that are frequently invisible. An important strength of this study is that it provides in-depth knowledge of private practice issues and reveals how dietitians nurture and protect the therapeutic relationship. The therapeutic relationship was shown to be an important learning environment and mediator of successful outcomes for patients, as well as crucial for a successful private practice business. Notably, the context of private practice was revealed as a stimulus for developing key strategies to nurture the therapeutic relationship. Further study in these areas would greatly benefit the area of dietetic private practice in Australia. The aspects of practice that contributed to successful outcomes for patients, such as empathy, perception, and reflective practice, are scarce in nutrition and dietetic studies. This study contributed important information on key strategies that are understudied, and that are poorly understood in the context of dietetic private practice.

This study used a lens of episteme, techne, and phronesis, to understand the different types of knowledge that dietitians use when performing key strategies and processes. In addition to using evidence and techniques that meet competency standards, other aspects of practice, such as, perception, intuition, empathy, and reflection in and on practice were evident, and as such, phronesis is a useful lens with which to view these aspects. In this regard, phronesis could add valuable dimensions to dietetic practice when gauging the differences between competent and expert dietitians. The framework of episteme, techne, and phronesis as knowledge types, provided a useful lens to explain a patient-centered holistic practice style and to examine how these key strategies are learnt and integrated into practice. This grounded theory study produced a model of private practice that shows concepts and relationships that could be explored quantitatively.

These strengths are countered by the fact that the practice and nutrition strategies were only investigated from the dietitian’s perspective. How patients experienced their care and approach was not a focus of this study, and there is a need for studies that consider the patients’ perspective of their care. Another limitation is that, although terms such as ‘client centred approach’, ‘mindfulness’, and ‘intuition’ have been used by the dietitians in this study, no further enquiry was made as to how similar in meaning these were to each other, when used by another dietitian. Due to the interview question, this model depicts what usually happens, from the dietitians view of a ‘case study’; that is, a long-term client. No data of how one-off ‘Medicare’ (public healthcare) patients were treated was included. Although the dietitians reported that these patients were treated similarly, the same outcomes were not seen with these short-term patients, due to the short length of care. The sample size (*n* = 11) was small; however, the sample represented a broad cross section of the profession and the concepts remained static across the sample, adding to the strength of the results. In this way, generality is plausible, but not possible with this sample size. Possibly due to the burden of the interview process on the participants, recruitment for this study was difficult, and the sample may be skewed towards dietitians who were interested in the subject matter and the trajectory of dietetics in Australia in general.

## 7. Conclusions

Dietetic private practice in Australia is a complex environment in which dietitians must employ high level professional, technical, and interpersonal skills. Private practice dietitians form collaborative relationships with their clients to nurture change over time and are able to provide continuity of care without departmental constraints. Thus, private practice is well placed to provide successful outcomes to those clients who can access long term private practice.

The context of private practice was revealed to be an important element of how and why dietitians develop and integrate knowledge over time. Further study aimed at uncovering more detailed information of how dietitians experience different aspects of private practice would be advantageous, to further develop our understanding of the challenges that dietitians face in private practice. The nuances that seem unique to private practice seem to facilitate a practitioner’s ability to respond in a client-centered manner, over a short period of time. Dietitians used intertwined forms of episteme, techne, and phronesis, to collaboratively nurture change in clients.

Episteme, techne, and phronesis offer a broader model of practice that has considerable applicability for nutrition and dietetics. Framing practice research within these concepts could be useful for addressing the complex aspects that are specific to the nutrition and dietetic profession.
